# Supplementary material for: Using Pose Estimation and 3D Rendered Models to Study Leg-Mediated Self-righting by Lanternflies
Source: Integr Comp Biol. 2024 Apr 10;64(3):776–94. doi: 10.1093/icb/icae014 (PMC11428466; doi:10.1093/icb/icae014)
Supplement: icae014_Supplemental_Files [file icae014_supplemental_files.zip › icb-2024-0030-File012.pdf]

Supplementary Materials

Using pose estimation and 3D rendered models to study leg-mediated self-righting by lanternflies

Theodore Bien[1§], Benjamin Alexander[1§], Chengpei Li[1§], Natalie Goeler-Slough[1], S. Tonia Hsieh[2], Suzanne Amador Kane [1*]

[1] Physics and Astronomy Department, Haverford College, 370 Lancaster Ave, Haverford, Pennsylvania, 19041 USA

[2] Department of Biology, BioLife Building, 1900 North 12th Street, Temple University, Philadelphia, 19122 USA

[§] Contributed equally

[*] Corresponding author

Email: samador@haverford.edu

Table of Contents

Supplemental Dataset S1 2

Movie 1.mp4 3

Fig. S1 6

Fig. S2 7

Fig. S3 8

Fig. S4 9

Fig. S5 11

Fig. S6 12

Fig. S7 14

Fig. S8 16

Fig. S9 17

Fig. S10 18

Fig. S11 19

Fig. S12 20

Fig. S13 21

Table S1. Summary statistics for terrestrial self-righting successful trials, times, attempt times and number of attempts per trial 22

Table S2. Results of linear regression on success rate, percent diagonal somersaulting, time/attempt and attempt number vs trial number 23

Table S3. Results of chi-squared proportion tests on the percent of successful self-righting attempts. 24

Table S4. Statistics from Kruskal-Wallis tests for self-righting times and attempt numbers for different life stages and substrates. 25

Table S5. Statistics from Kruskal-Wallis tests for self-righting times and attempt numbers for different righting methods. 26

Table S6. Similarity values from cross-correlation analysis of tarsal z motion in the body frame 27

Supplemental Dataset S1

Dataset S1.zip: Datasets and computer code used for analysis and creating models and figures

Movie 1.mp4

Videos showing examples of the behaviors used by spotted lanternfly nymphs during successful and failed terrestrial self-righting attempts, and animations of the 3D models.

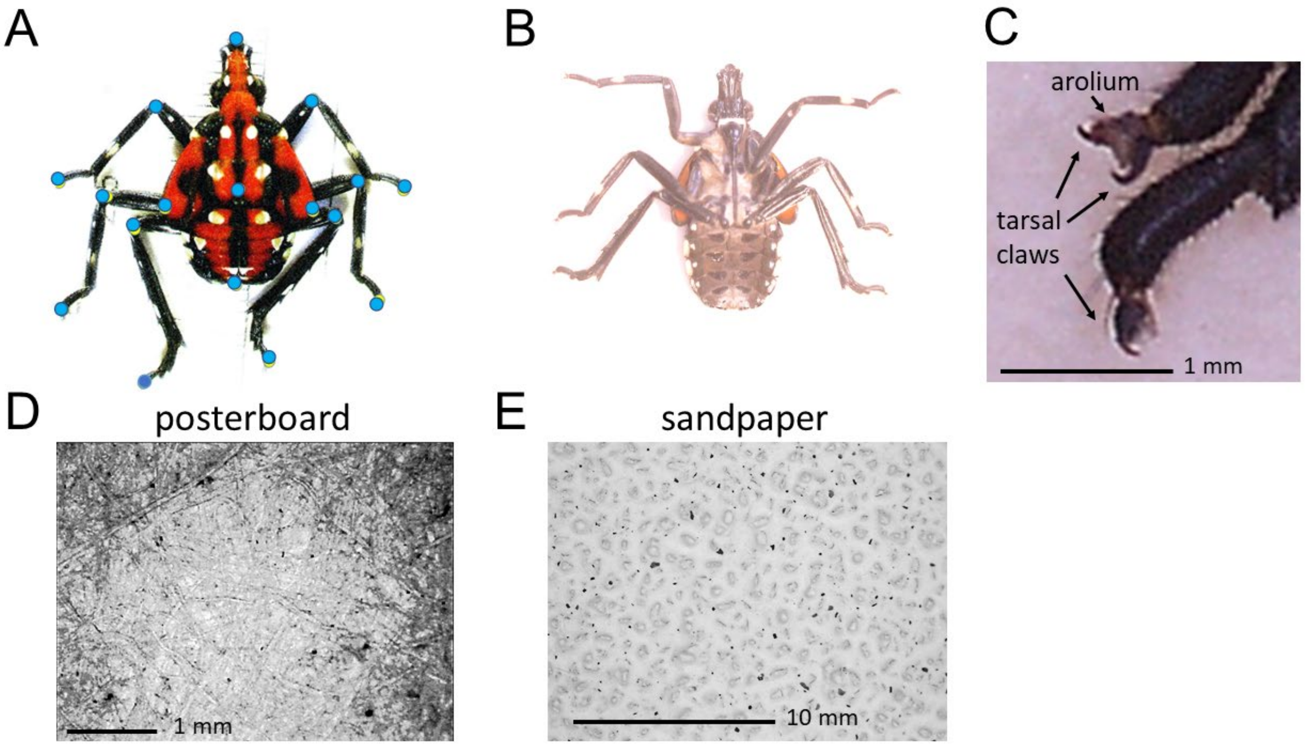

Fig. S1

A) Top view of a spotted lanternfly nymph showing the landmarks used for tracking the body pose on video, including the cranial and caudal endpoints, right and left edges of the wingbuds on the ventral surface, and 2 joints on each leg, for a total of 16 points tracked on at least 2 views in almost all video frames. A point on the dorsal and ventral surface was tracked where possible. B) Ventral view showing the articulation of the legs and their points of insertion on the ventral surface. C) Micrograph of the tarsal claws on two feet and the arolium (adhesive pad located in between the pair of claws at top).D-E) Micrographs of the posterboard and sandpaper substrates used for self-righting experiments.

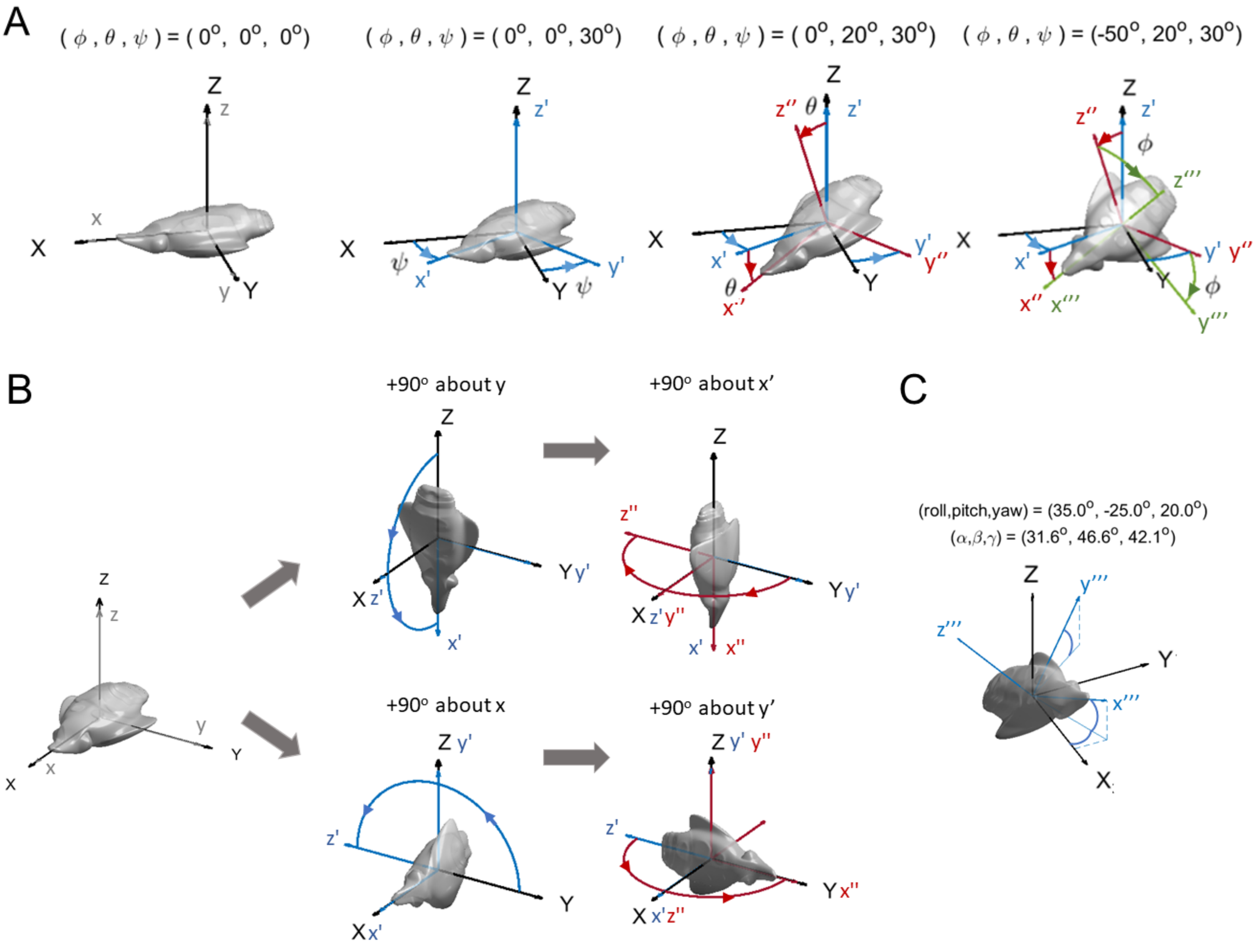
A
(ϕ , θ , ψ) = (0°, 0°, 0°)
(ϕ , θ , ψ) = (0°, 0°, 30°)
(ϕ , θ , ψ) = (0°, 20°, 30°)
(ϕ , θ , ψ) = (-50°, 20°, 30°)
B
+90° about y
+90° about x'
+90° about x
+90° about y'
C
(roll,pitch,yaw) = (35.0°, -25.0°, 20.0°)
(α,β,γ) = (31.6°, 46.6°, 42.1°)

Fig. S2

A) Illustration of the three successive yaw-pitch-roll rotations, where the axes' orientations are shown as *x'y'z', x"y"z" and x'"y'"z'"* after the first, second and third rotations, respectively. B) Example image showing that performing the 3D rotations denoted by the Euler angles in different order result sin different final orientations. More generally, rotations in 3D do not commute: i.e., rotations about different axes that occur in a different order result in different final 3D orientations. C) Illustration of how the angles that describe the final orientation of the body *xyz* axes with respect to the *XYZ* axes of the spatial frame (i.e., the angle α between *x'"* and *X*, β between *y'"* and *Y*, and γ between *z*''' and *Z*) do not in general correspond to the roll, pitch and yaw Euler angles. (Figures created with code adapted from MATLAB example code (e.g. *dr.draw3DOrientation* at https://www.mathworks.com/help/nav/ug/rotations-orientation-and-quaternions.html, accessed January 16, 2024).

Fig. S3

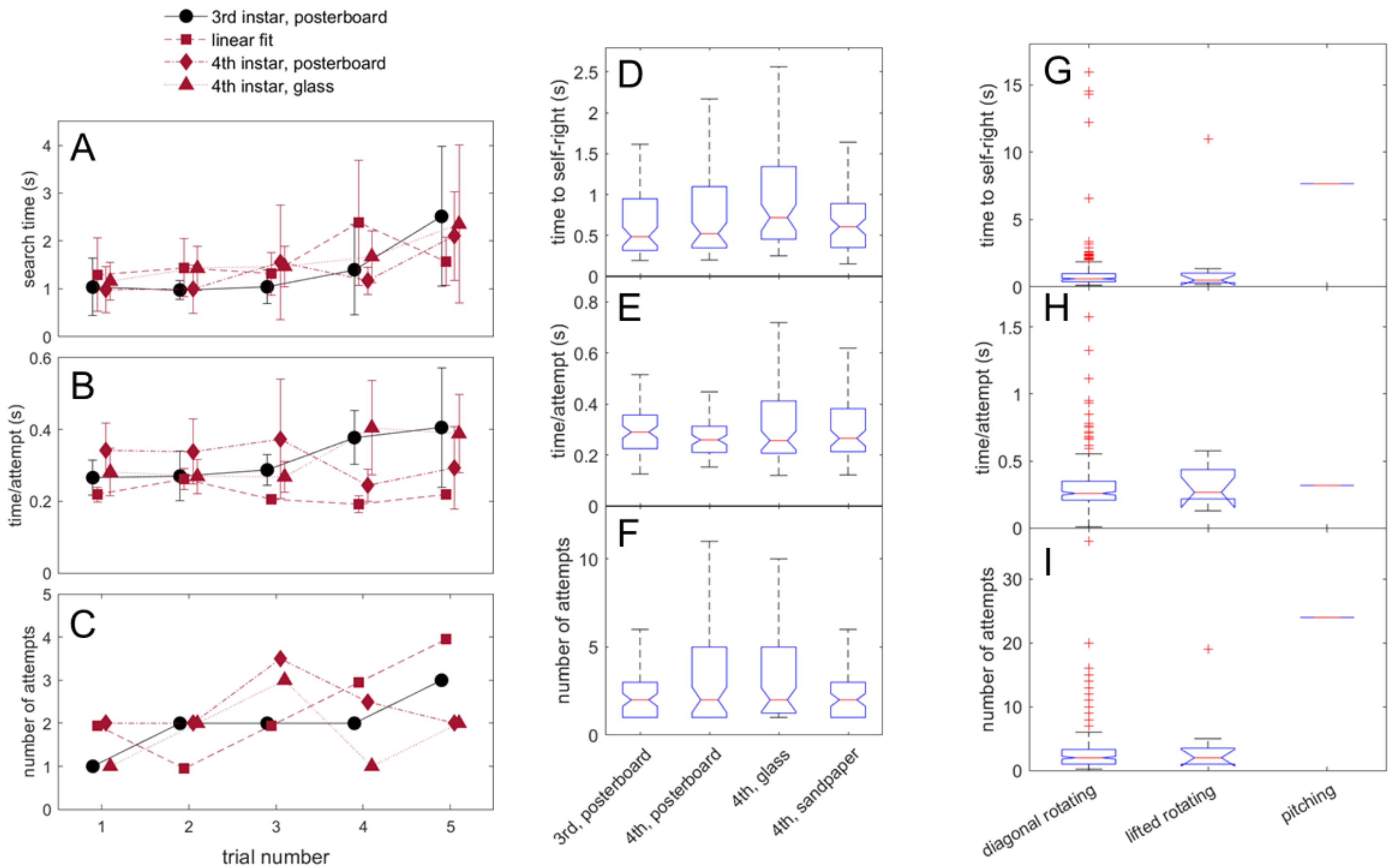

Dependence on trial number of the time spent searching before attempting righting (A), time/attempt (B), and (C) number of attempts during terrestrial self-righting, broken down by life stage and substrate. Error bars in A and B indicate 95% CI. Box-and-whisker plots of (D) time to self-right, (E) time/attempt and (F) number of attempts vs substrate and life stage combinations. (G-I) show the same quantities vs righting methods. In D-I, the red lines are medians, blue boxes the 25th to 75% percentiles, outliers are red + markers, and whiskers denote maximum and minimum values that are not outliers. If the notches around the medians of each pair of data fail to overlap, then the medians disagree at the 95% CI.

Fig. S4

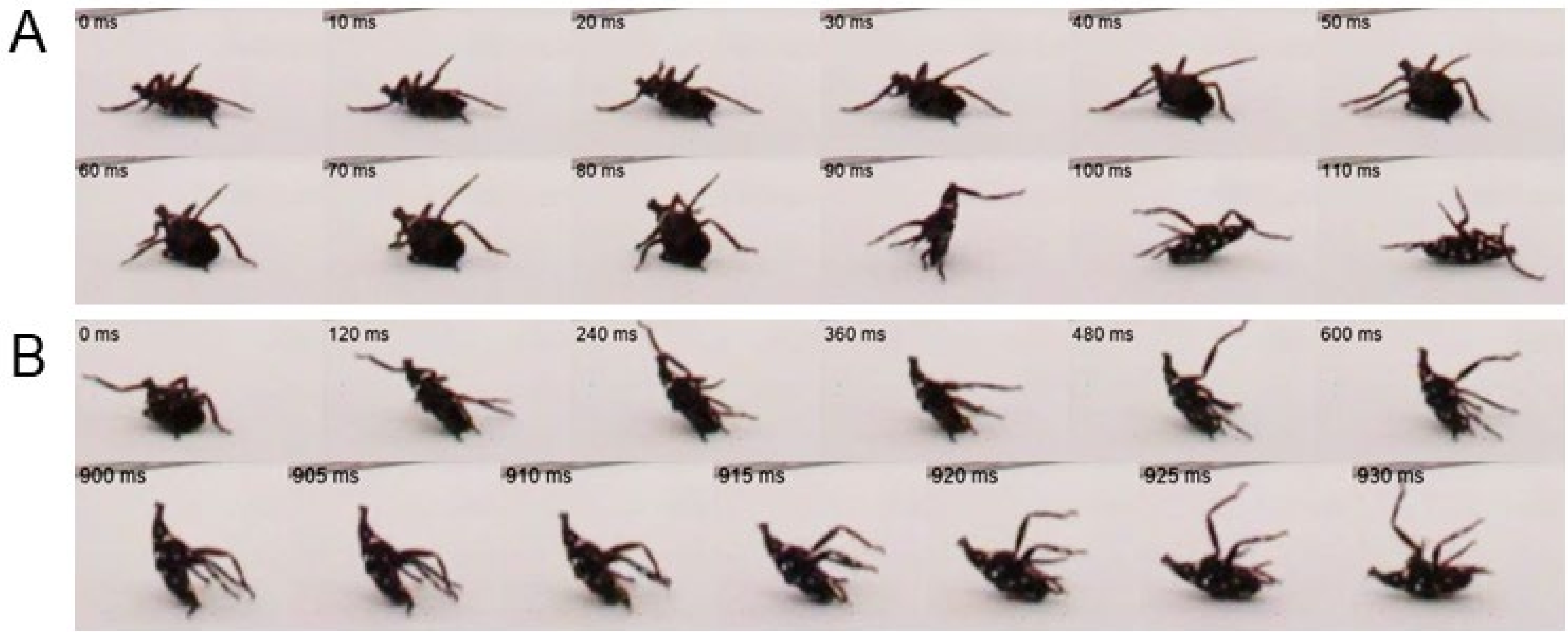

Sequences of closeup still images (times relative to the first image shown at top left; spacings between frames also indicated in caption) for attempts by 3rd instar spotted lanternfly nymphs to right on posterboard that failed because of A) slipping (every 20 ms) and B) inadequate torque (top row: every 120 ms; bottom row: every 5 ms).

Fig. S5

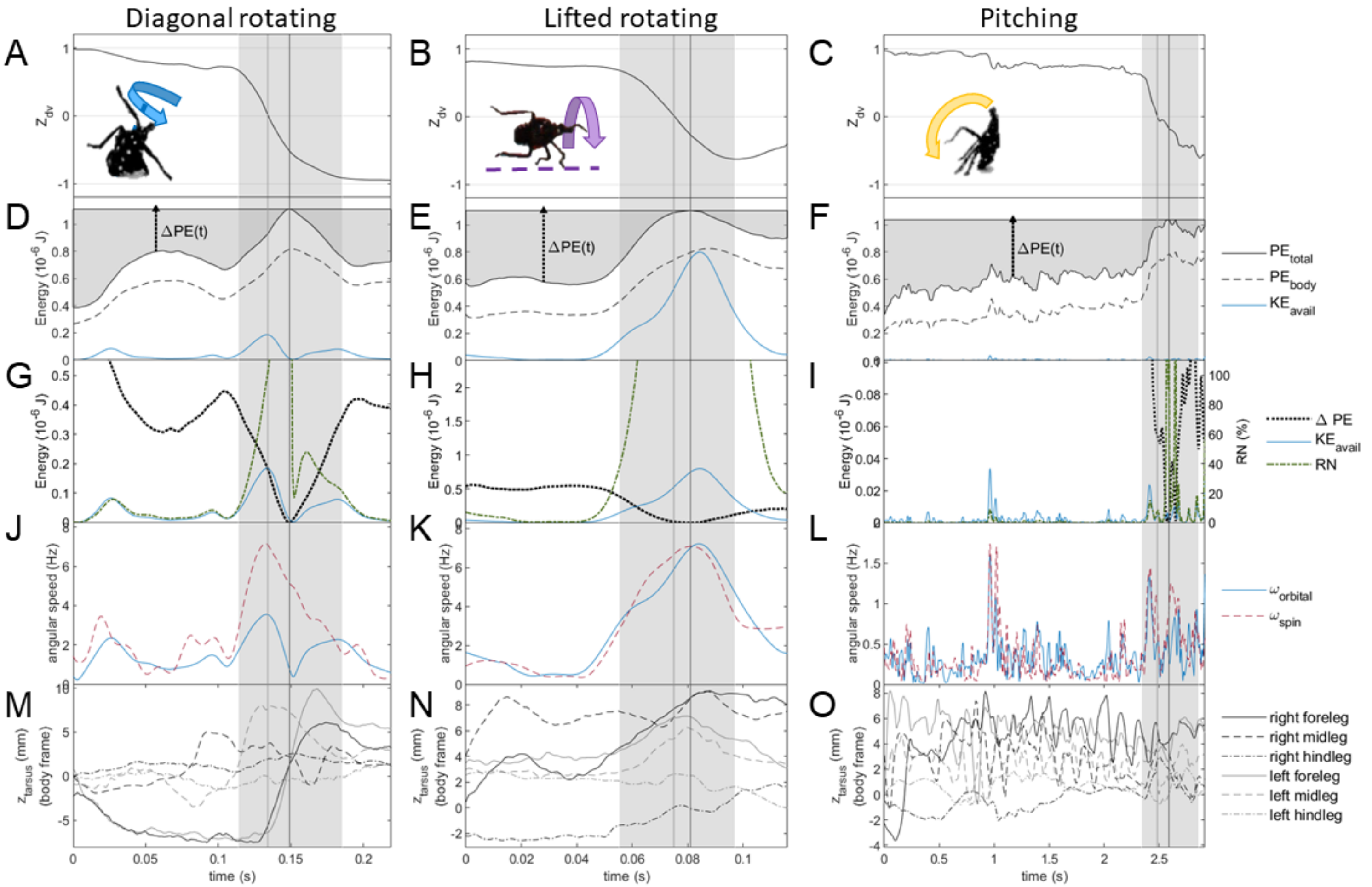

Additional results of analysis of the tracked coordinates and 3D model plotted for diagonal rotating and lifted rotating not shown in Fig. 5, and full results for pitching. Note that different time intervals were plotted for each righting mode for diagonal rotating and lifted rotating to display data for approximately equal relative time intervals before and during active overturning (gray shaded region) (A-C) show the z-component of the dorsal-ventral axis, which transitions from ≈ +1 when overturned to 0 at the flipping point to a final value ≥ -1 with the body pitched upward in the resting pose. (D-F) show the time behavior of gravitational potential energy of the whole insect and its body, and available kinetic energy, K_{avail}, computed

from the inverted physical pendulum model (see text for details). These plots also illustrate the increase in potential energy, *ΔPE(t)*, required to right at any given time. The plots in (G-I) compare the time dependence of *ΔPE(t),* K_{avail} and the righting number, *RN*, used to characterize the contribution of inertial reorientation to righting. Note especially that the energy scale for pitching in (I) is approximately an order of magnitude lower than that for the other two methods. The plots in (J-L) compare the time behavior of the angular velocity of the body's spin motion along its pitch and roll axes with the orbital angular velocity computed from the inverted physical pendulum model; see Fig. 3F for the definitions of $\omega_{orbital}$ and ω_{spin}; note that ω_{spin} only includes contributions from roll and pitch rotations. (M-O) show the motion of the tarsi (feet) along in the z-axis in the body frame, as defined in Fig. 1E. The light gray vertical line denotes the flipping point (Z_{dv} = 0) when the specimen transitions from overturned, and the dark gray vertical line the apex of its trajectory when total PE is at its maximum. (Fig. 1I-K)

Fig. S6

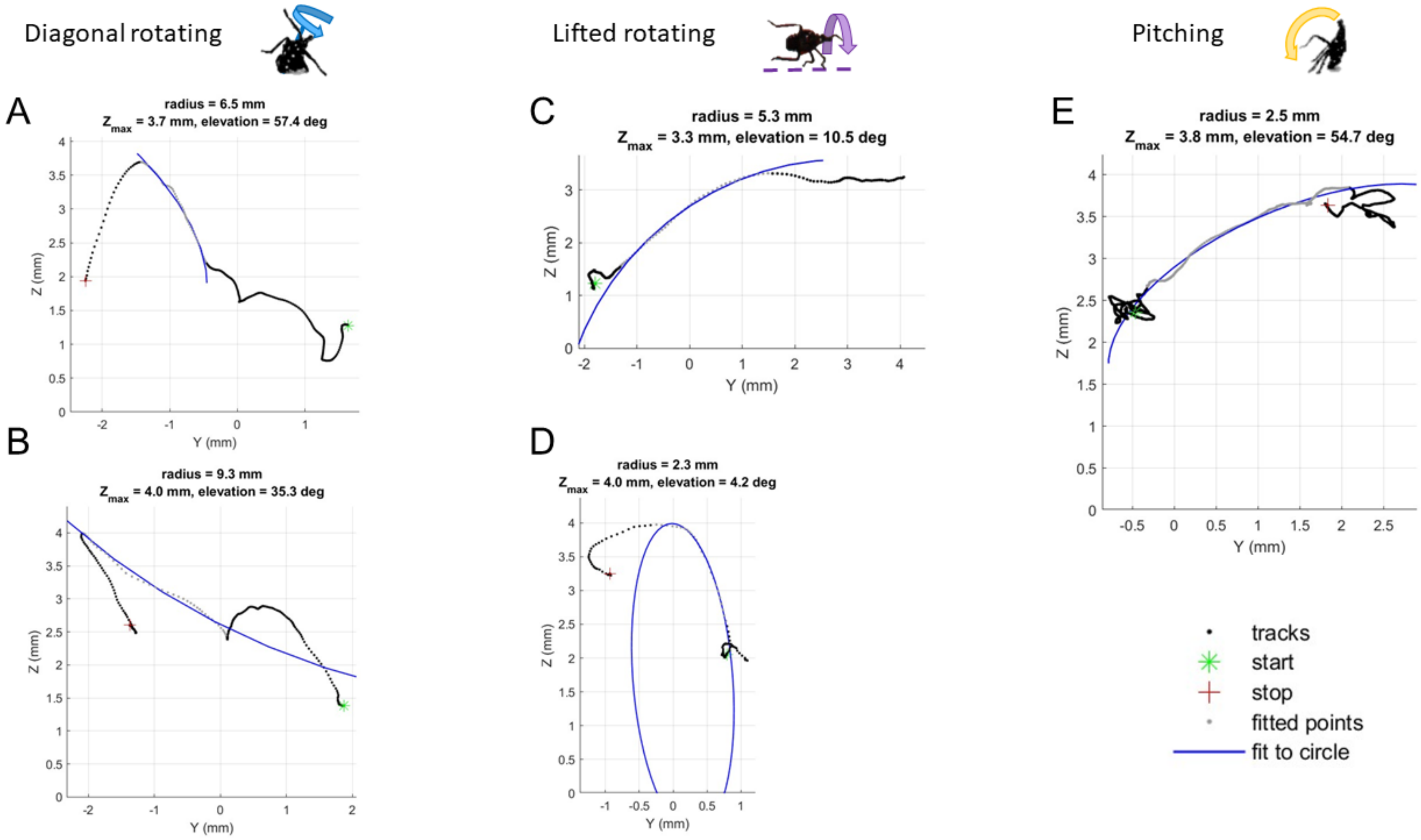

Insect center of mass (COM) trajectory during self-righting compared to a fit to a circle from the start of active overturning the apex (maximum height) of the trajectory. In each case, the maximum fit residual was less than the tracking uncertainty. Note that the fit in (B) does not correspond to the correct curvature to agree with geometry predicted by the inverted pendulum model

Fig. S7

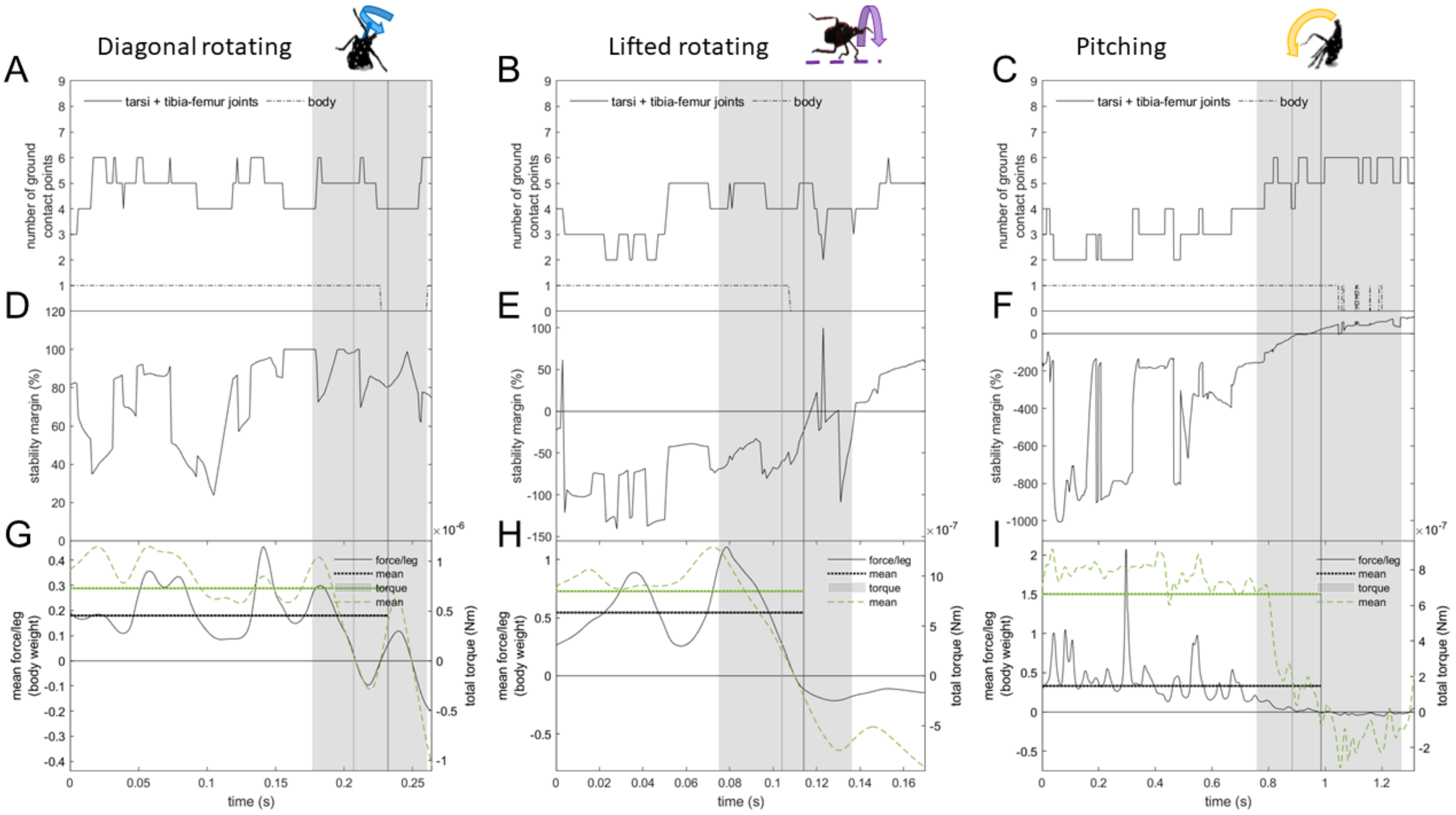
Diagonal rotating
Lifted rotating
Pitching
A
B
C
D
E
F
G
H
I
tarsi + tibia-femur joints
body
number of ground contact points
stability margin (%)
mean force/leg (body weight)
total torque (Nm)
force/leg
mean
torque
mean
time (s)

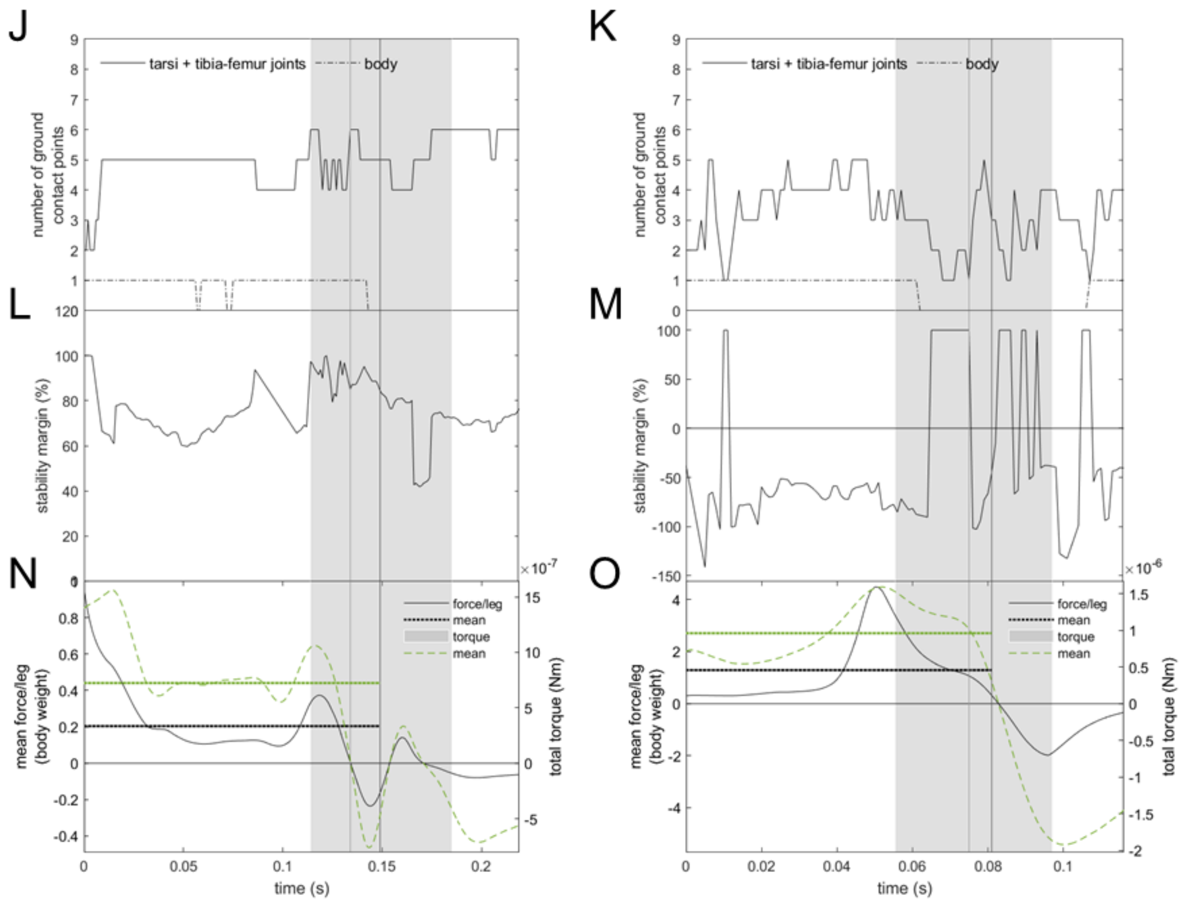

(A, B, C, J, K) number of leg and body points in contact with the ground, (D, E, F, L, M) stability margin/ideal stability margin (SM %) computed as percent ideal stability margin, (G, H, I, N, O) ground reaction force per leg and total torque vs time. Note that the abrupt changes in the stability margin and force per leg are due to changes in the number of leg parts contacting the ground. The light gray vertical line denotes the flipping point when the specimen transitions from overturned, and the dark gray vertical line the apex of its trajectory, while the shaded gray region denotes active overturning.

Fig. S8

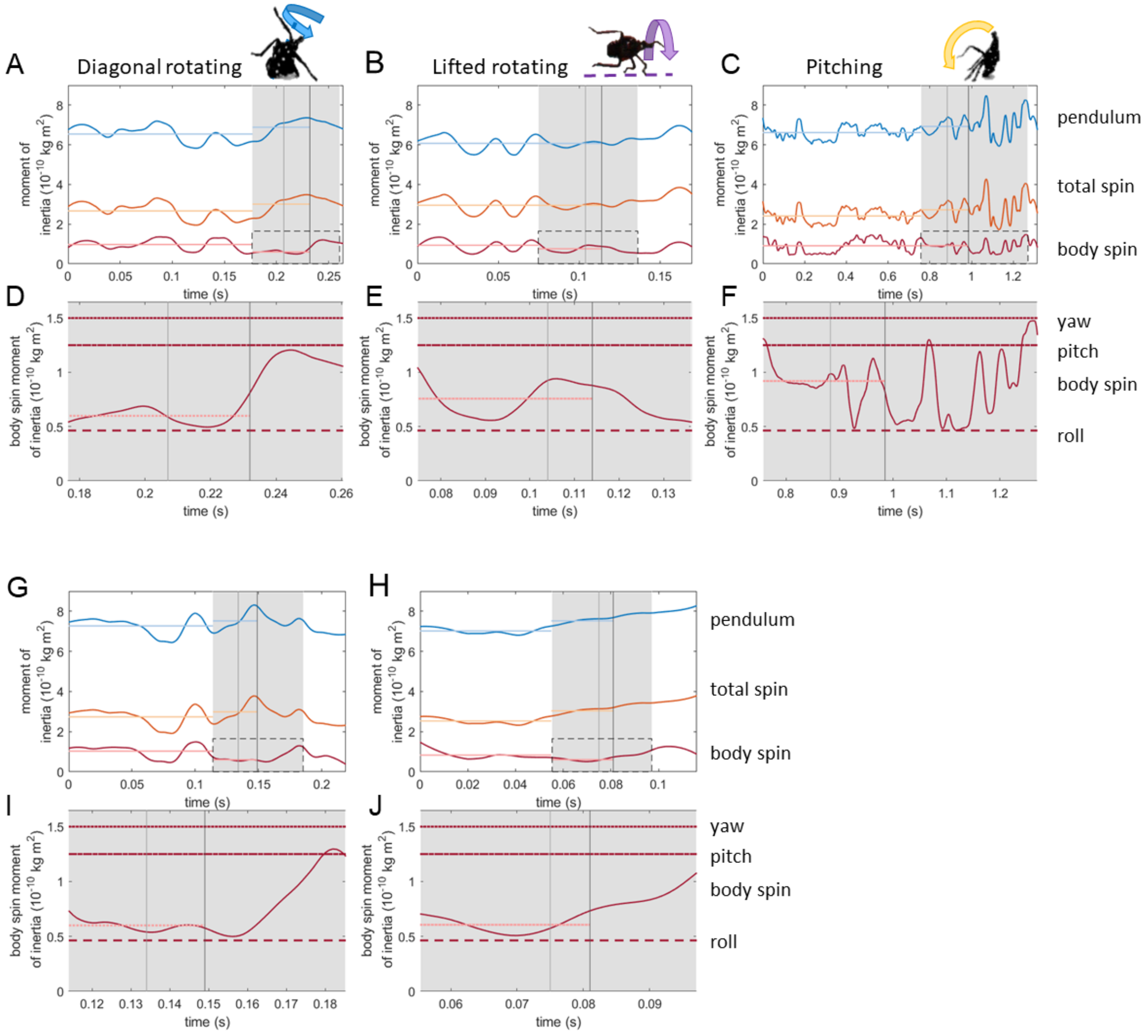

Moment of inertia values for the three rotational modes used by spotted lanternfly nymphs for self-righting. (A, B, C, G, H) Moment of inertia vs time immediately before and during righting for the physical pendulum model, $I_{pendulum}$, the whole insect spin moment of inertia including body and legs, $I_{spin,total}$, the spin moment of inertia of only the body, I_{body}, and the mean value of I_{body}. (D,E,F,I,J) Zoomed in plot of the region shown in the gray dashed boxes in A-C that shows the relationship of the body's spin moment of inertia, I_{body}, to its mean and to the values for pure roll, pitch and yaw rotations. These values correspond to a body spin rotation axis [roll, pitch, yaw] of [0.94, -0.17, 0.29] ± 0.02 for diagonal rotating, [-0.89,0.43,-0.13] ± 0.02 for lifted rotating, and [0.61, -0.77, 0.17] ± 0.02 for pitching, equivalent to the angle, δ, between the body's spin rotation axis & cranial-caudal axis (Fig. 3H) being equal to 18 ± 3°, 28 ±

11 °, and 50 ± 8 °, respectively. The light gray vertical line denotes the flipping point when the specimen transitions from overturned, and the dark gray vertical line the apex of its trajectory, while the shaded gray region denotes active overturning.

Fig. S9

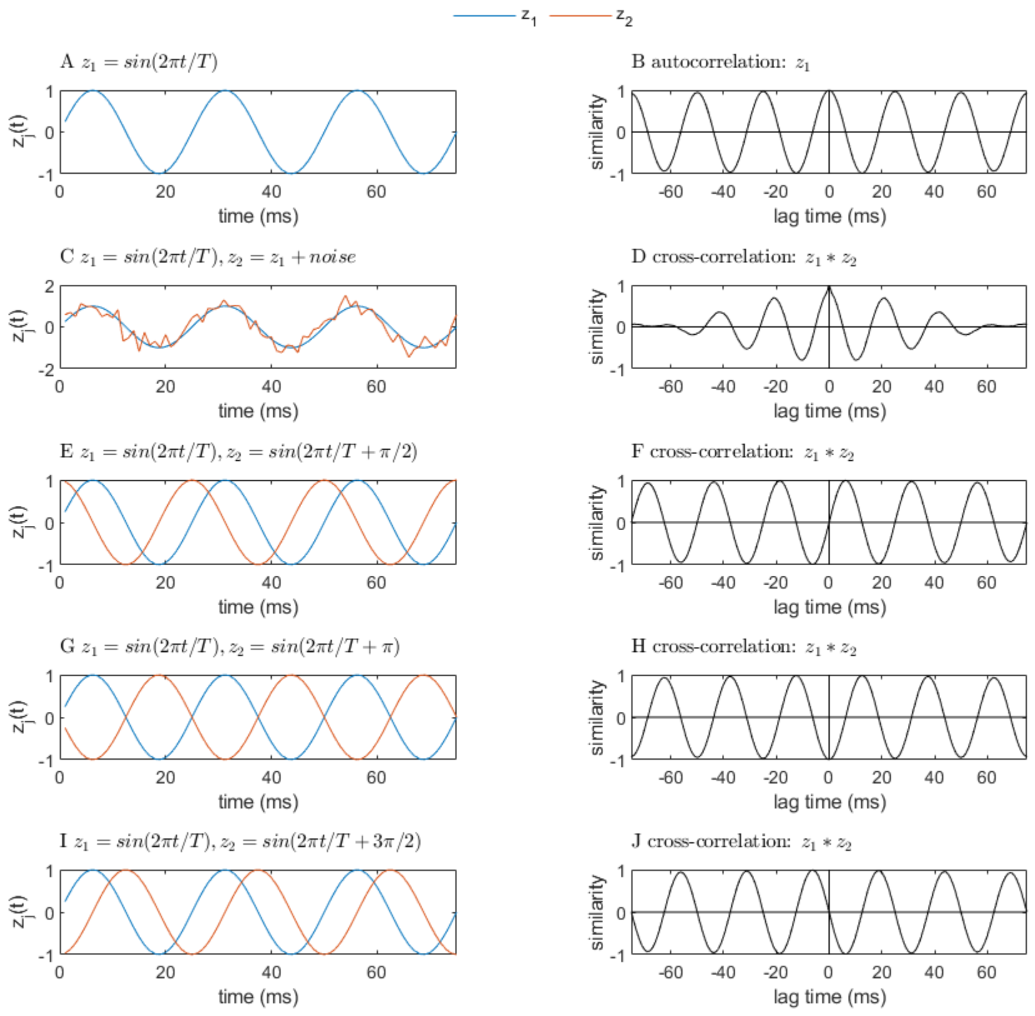

Illustrative examples of the cross-correlation vs lag time (time offset) useful in interpreting the results in Fig. S7-9. A, B) The normalized autocorrelation (cross-correlation of a function with itself) for a sine function, $z_1 = \sin(2\pi t / T)$, where the period T = 25 ms, has value 1 at zero lag time by definition, and peaks at integer multiples of the period. C, D) The cross-correlation of the sine function from A) with the sine function with noisy period and amplitude, still has periodic peaks, but their amplitude decreases to zero as absolute lag time increases. E-I) illustrate the cross-correlation of the sine function from A) with another sine with the same period, T, but a nonzero phase offset, φ: $z_2 = \sin(2\pi t / T + \varphi)$. The following plots show the cross-correlation between a sine and a sine with the same period with a phase offset of: B) $\varphi = 0$ (in phase), F) $\varphi = \pi/2$, H) $\varphi = \pi$ (out of phase), and J) $\varphi = 3\pi/2$.

Fig. S10

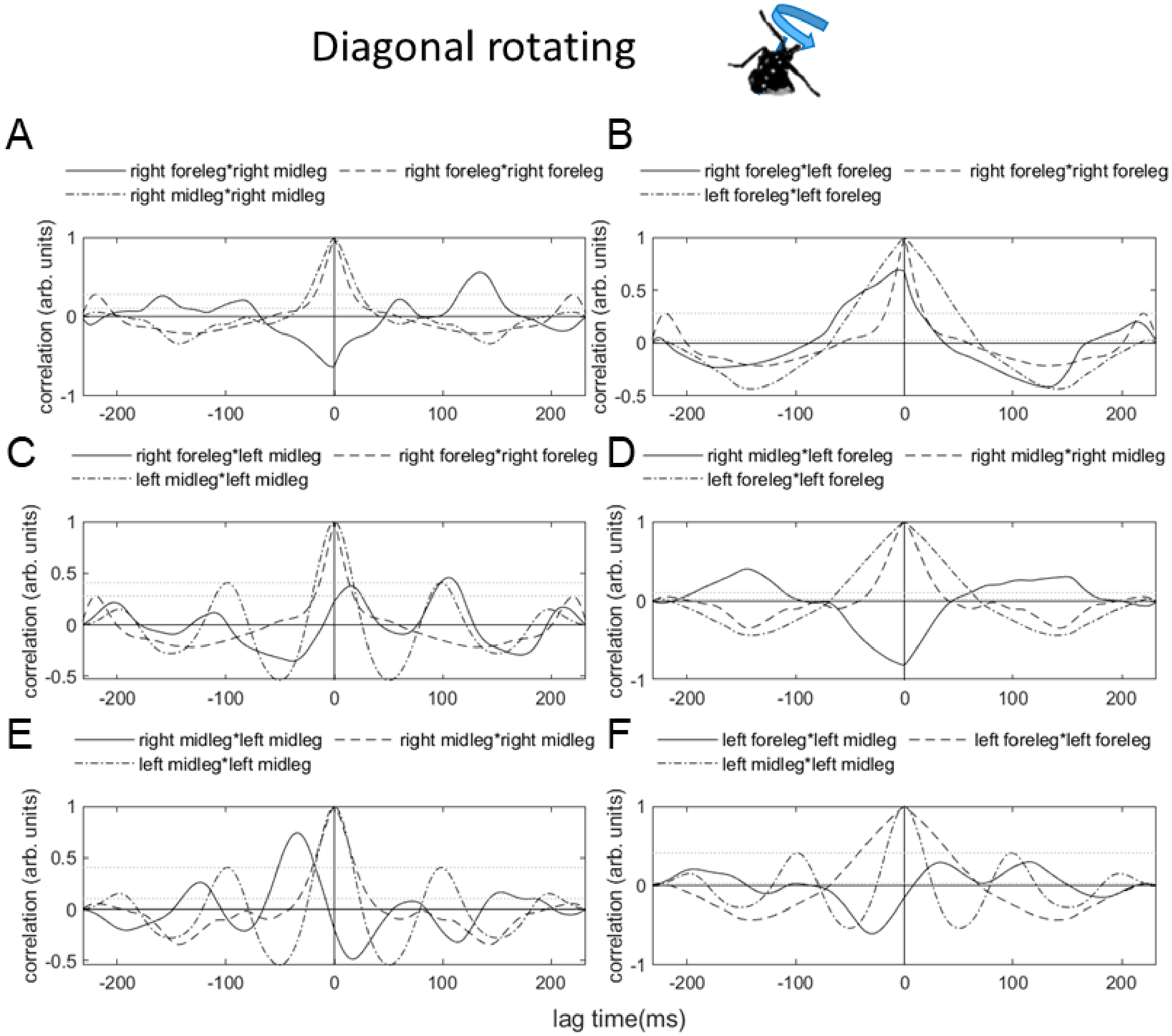

Cross-correlation of each pair of legs (solid lines) compared with the corresponding autocorrelation for each leg in the pair (dashed and dot-dashed line) during righting by diagonal rotating. Note that the right legs are uppermost during righting in this trial, while the legs on the left side primarily support the body. (Gray horizontal lines indicate the maximum values of the autocorrelation for each leg in the pair at nonzero lag time.)

Fig. S11

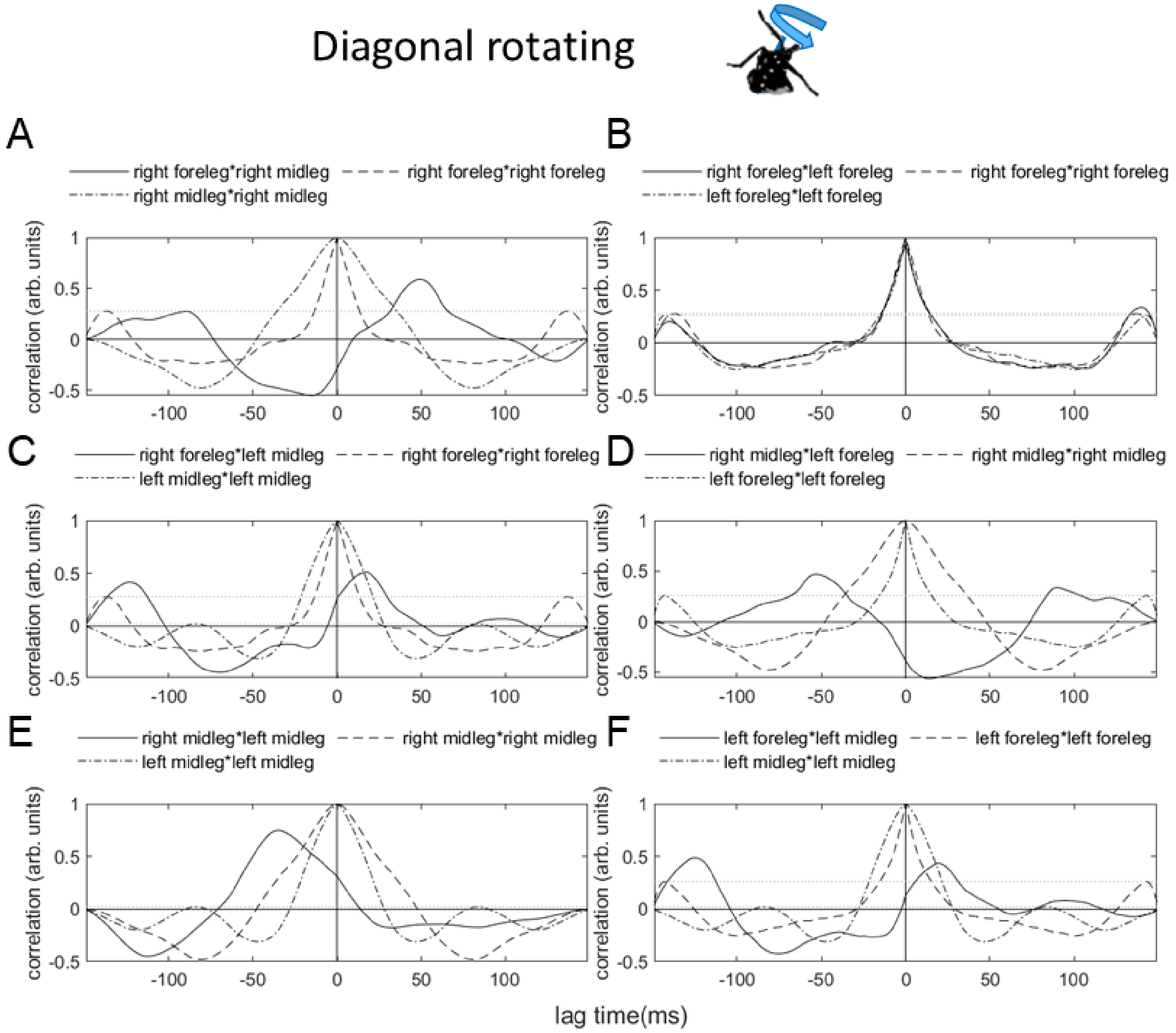

See caption for Fig. S9

Fig. S12

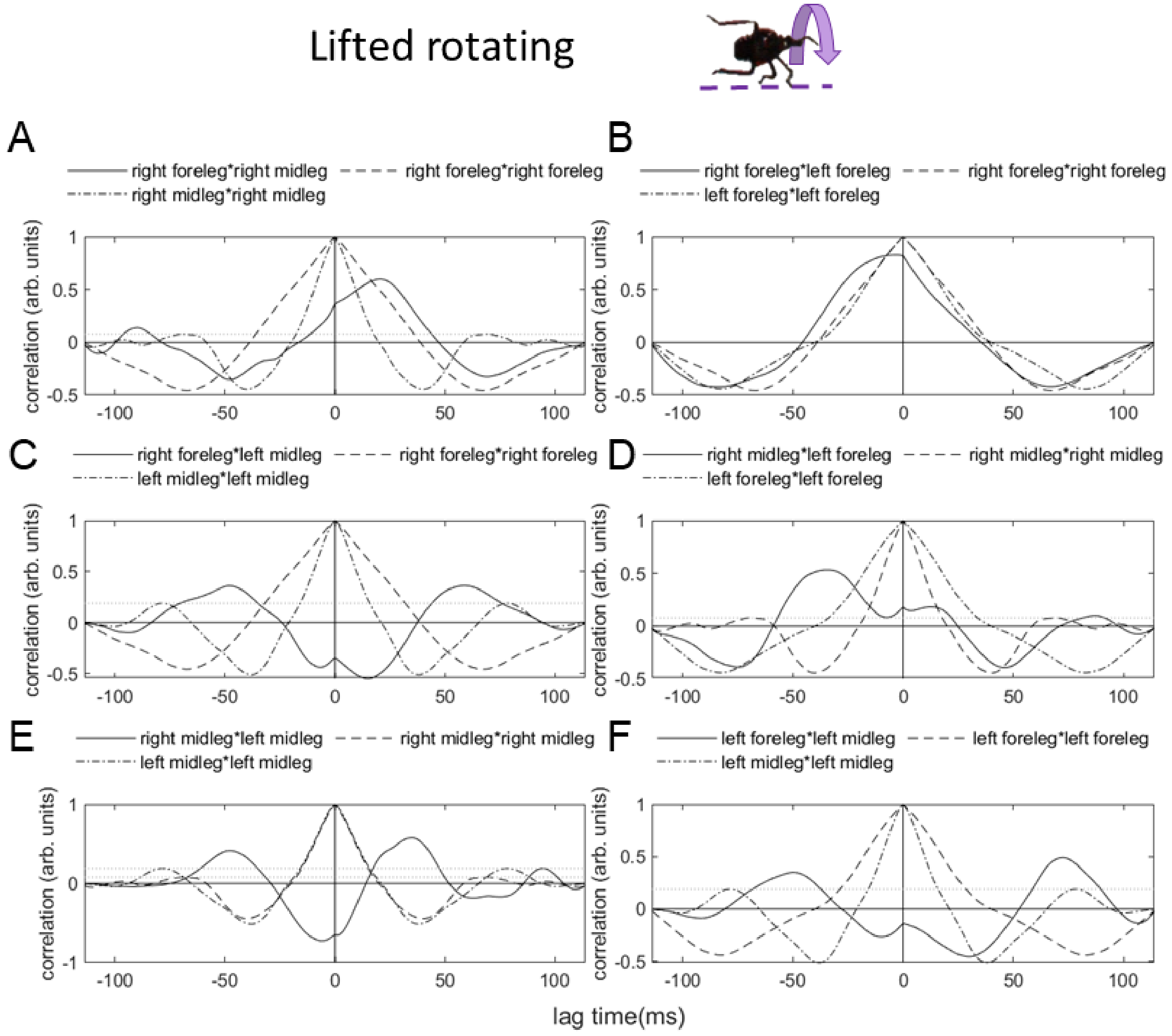

See caption for Fig. S9

Fig. S13

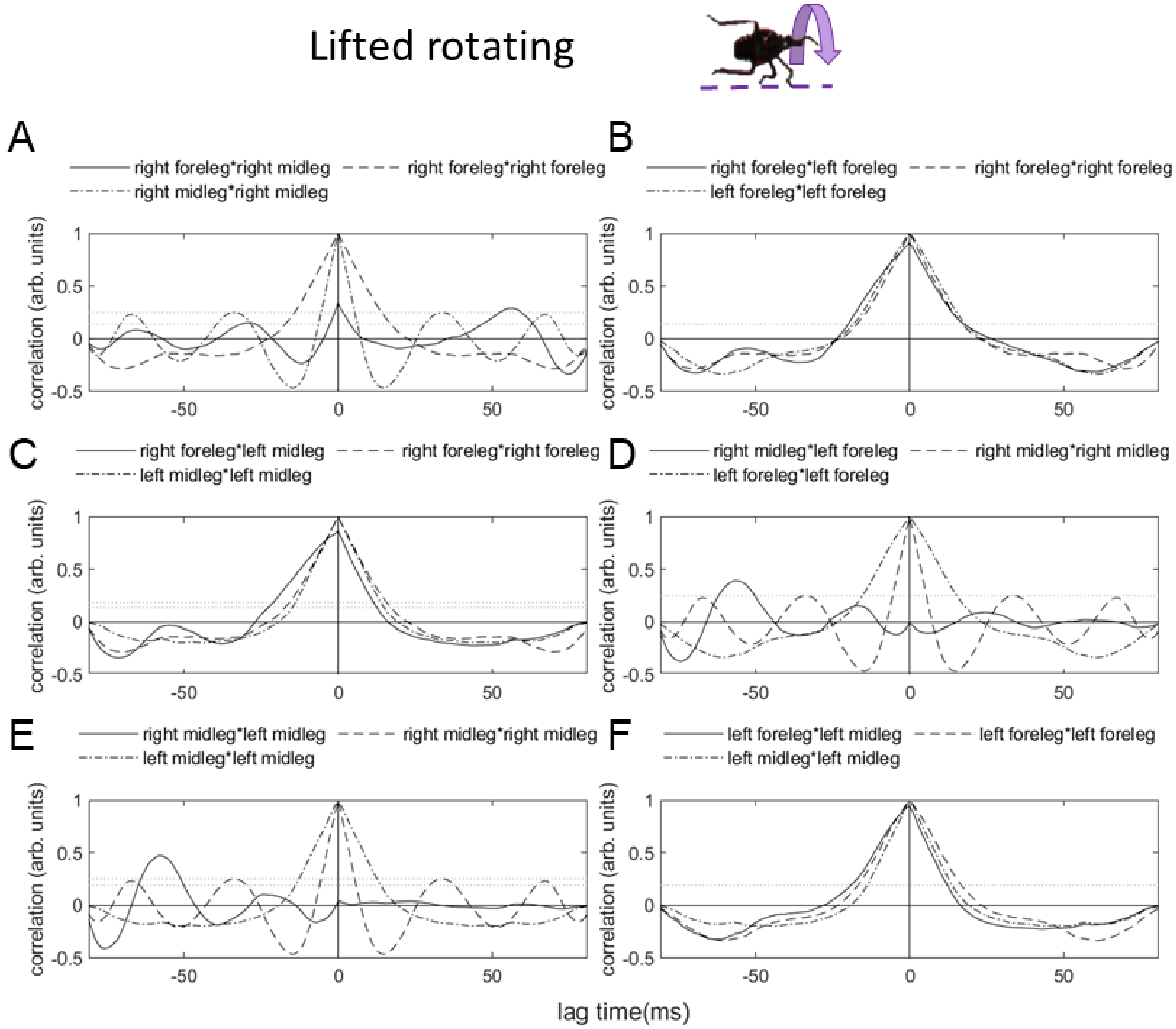

See caption for Fig. S9

Fig. S14

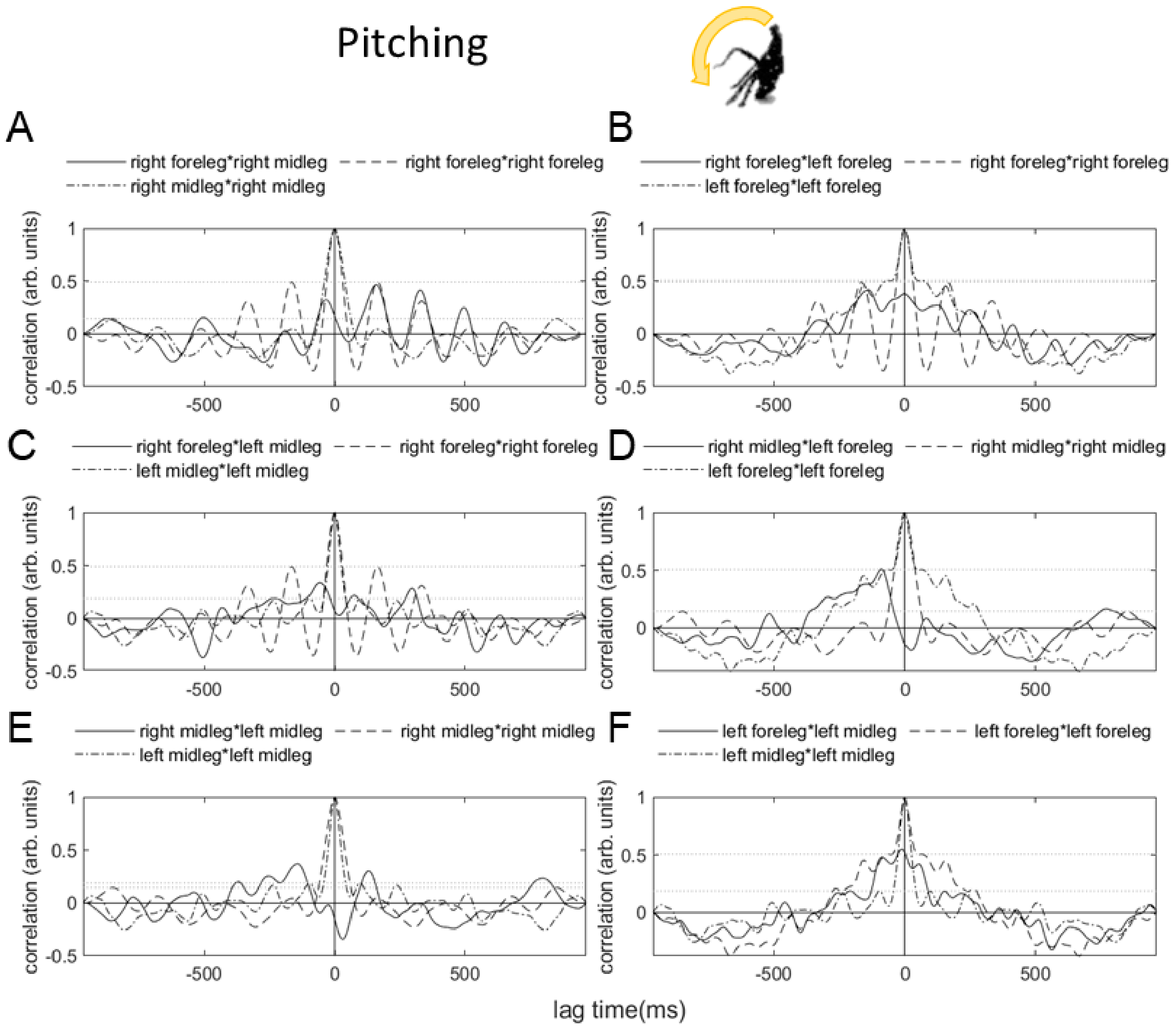

See caption for Fig. S9

Table S1. Summary statistics for terrestrial self-righting successful trials, times, attempt times and number of attempts per trial

Data for times and number of attempts are given as median ± MAD, where MAD = median absolute deviation.

Substrate	Instar	Number of trials	Number (%) successful trials	time to self-right (ms)	attempt time (ms)	number of attempts
posterboard	3rd	75	75 (100%)	486 ± 228	290 ± 65	2 ± 1
posterboard	4th	75	70 (93%)	517 ± 206	255 ± 45	2 ± 1
glass	4th	75	69 (92%)	687 ± 278	253 ± 69	2 ± 1
sandpaper	4th	74	74 (100%)	607 ± 255	266 ± 79	2 ± 1

Table S2. Results of linear regression on success rate, percent diagonal somersaulting, time/attempt and attempt number vs trial number

Sample size n = 15. Degrees of freedom = 3. * Results for the 3rd instar self-righting success rate on posterboard are not reported because they were 100% for all trials.

Self-righting success rate

Life stage, substrate	R-squared	F-statistic	P-value
3rd instar, posterboard *	---	---	---
4th instar, posterboard	0.63	5.0	.11
4th instar, glass	0.59	4.4	.13
4th instar, sandpaper	0	0	1

Time/attempt

Life stage, substrate	R-squared	F-statistic	P-value
3rd instar, posterboard	0.68	6.4	0.09
4th instar, posterboard	0.30	1.3	0.34
4th instar, glass	0.64	5.3	0.11
4th instar, sandpaper	0.36	1.7	0.28

Attempt number

Life stage, substrate	R-squared	F-statistic	P-value
3rd instar, posterboard	0.80	12	0.04
4th instar, posterboard	0.69	6.8	0.08
4th instar, glass	0.01	0.045	0.85
4th instar, sandpaper	0.04	0.11	0.76

Table S3. Results of chi-squared proportion tests on the percent of successful self-righting attempts.

Group A, B = instar, substrate. Sample size: n = 75 for all conditions except 4th instars on sandpaper for which n = 74. Degrees of freedom = 1.

Group A	Group B	p-value	chi-squared
3rd/posterboard	4th/posterboard	0.07	3.3
4th, posterboard	4th, glass	0.73	0.11
4th, posterboard	4th, sandpaper	0.07	3.3
4th, glass	4th, sandpaper	0.07	3.3

Table S4. Statistics from Kruskal-Wallis tests for self-righting times and attempt numbers for different life stages and substrates.

Group A, B = instar, substrate. Sample size: n = 75 for all conditions except 4th instars on sandpaper for which n = 74. Limits are used for testing but are not simultaneous confidence intervals.

Time to self-right

Group A	Group B	Lower Limit	A-B	Upper Limit	P-value
3rd, posterboard	4th, posterboard	-43.2	-6.9	29. 4	0.96
4th, posterboard	4th, glass	-65.3	-29.0	7.3	0.17
4th, posterboard	4th, sandpaper	-30.4	6.0	42.4	0.97
4th, glass	4th, sandpaper	-1.4	35.0	71.4	0.065

Time/attempt

Group A	Group B	Lower Limit	A-B	Upper Limit	P-value
3rd, posterboard	4th, posterboard	-20.3	16.0	52.2	0.67
4th, posterboard	4th, glass	-48.7	-12.5	23.8	0.81
4th, posterboard	4th, sandpaper	-48.9	-12.5	23.9	0.82
4th, glass	4th, sandpaper	-36.4	0.023	36.4	1

Number of attempts

Group A	Group B	Lower Limit	A-B	Upper Limit	P-value
3rd, posterboard	4th, posterboard	-54.4	-19.1	16.1	0.50
4th, posterboard	4th, glass	-42.3	-7.0	28.2	0.96
4th, posterboard	4th, sandpaper	-16.1	19.2	54.6	0.50
4th, glass	4th, sandpaper	-9.1	26.3	61.6	0.22

Table S5. Statistics from Kruskal-Wallis tests for self-righting times and attempt numbers for different righting methods.

Group A, B = two different righting method. Sample size: n = 289 for diagonal rotating, 9 for lifted rotating and 1 for pitching. Limits are used for testing but are not simultaneous confidence intervals.

Time to self-right

Group A	Group B	Lower Limit	A-B	Upper Limit	P-value
Diagonal righting	Lifted righting	-55.583	11.426	78.435	0.91574

Time/attempt

Group A	Group B	Lower Limit	A-B	Upper Limit	P-value
Diagonal righting	Lifted righting	-74.376	-7.3664	59.643	0.96407

Number of attempts

Group A	Group B	Lower Limit	A-B	Upper Limit	P-value
Diagonal righting	Lifted righting	-62.248	2.7553	67.759	0.99457

Table S6. Similarity values from cross-correlation analysis of tarsal z motion in the body frame

Here these values correspond to the maximum similarity for each pair of legs in Fig. S7-9; note that 1 = perfect correlation, 0 = no correlation.

**Righting mode**	**Diagonal rotating trial 1, trial 2**	**Lifted rotating trial 1, trial 2**	**Pitching**
**Leg pair**	**Maximum similarity**		
right foreleg*right midleg (ipsilateral pair)	0.62, 0.59	0.60, 0.34	0.47
left foreleg*left midleg (ipsilateral pair)	0.61, 0.94	0.49, 0.92	0.55
right foreleg*left foreleg (contralateral pair)	0.69, 0.51	0.83, 0.87	0.42
right midleg*left midleg (contralateral pair)	0.74, 0.56	0.73, 0.40	0.37
right foreleg*left midleg	0.46, 0.75	0.55, 0.47	0.37
right midleg*left foreleg	0.81, 0.49	0.53, 0.95	0.51
